# A Cross-Sectional Study of Price and Affordability of Drugs for Rare Diseases in Shandong Province, China

**DOI:** 10.3390/ijerph192013319

**Published:** 2022-10-15

**Authors:** Yan Mu, Kuimeng Song, Yan Song

**Affiliations:** 1 School of Healthcare Security, Shandong First Medical University & Shandong Academy of Medical Sciences, Jinan 250117, China; 2 Shandong Institute of Medicine and Health Information, Jinan 250117, China

**Keywords:** rare diseases, rare disease drugs, medicine prices, affordability, health insurance, China

## Abstract

Objective: The affordability of rare disease drugs has become a social issue that cannot be ignored. This study aims to evaluate the current price and affordability of rare disease drugs in China, with evidence from Shandong province. Methods: Data on prices and affordability of 50 drugs for 22 rare diseases were collected from secondary and tertiary public hospitals in Shandong Province, using an adaptation of the World Health Organization/Health Action International (WHO/HAI) methodology. Prices were measured as Median Price Ratios (MPRs). Affordability was measured as days of daily per capita disposable income required for the cost of one month’s treatment. Results: Out of the 50 rare disease drugs, 11 drugs had MSH reference prices and 34 had PBS reference prices. Median prices of 11 drugs were higher than MSH reference prices (median 1.33), and median prices of 34 drugs were higher that Australian PBS prices (median 1.97). Thirty-six (72.00%) and forty-four (88.00%) drugs were unaffordable for urban and rural residents, respectively. Thirty-four (68.00%) and thirty-eight (76.00%) drugs were unaffordable for urban and rural residents even after reimbursement by the health insurance schemes of China, respectively. Conclusions: The affordability of some rare disease drugs remained poor with their relatively high prices in Shandong Province. Sustainable mechanisms are needed to reduce the price of rare disease drugs and to improve the affordability of rare disease patients.

## 1. Introduction

A rare disease is any disease with a low prevalence that affects a limited number of individuals but is potentially complex, chronic, progressive, degenerative, and often life-threatening [1,2]. There is no unified standard for rare diseases and the definition differs across countries [3]. A disease or disorder is defined as rare when it affects no more than 1 in 1250 individuals in the USA [4], no more than 1 in 2000 individuals in European Union [2], and no more than 1 in 2500 people in Japan [5]. There is still a lack of sufficient epidemiological data on rare diseases and no clear official definition in terms of prevalence in China. It is estimated that there are more than 16.8 million people with rare diseases in China, based on China’s large population [6]. Patients with rare diseases face a much heavier economic burden due to the high price of drugs for rare diseases [7,8]. Attention to rare disease drugs and the social security system for rare diseases in China is of great significance to protect the rights of patients with rare diseases and safeguard social fairness and justice.

As society’s attention to rare diseases has increased, China has recently issued several policies to improve the access to drugs for rare diseases. In 2018, China’s First National Rare Disease List involving 121 diseases was issued jointly by five national ministries [9]. The list of rare diseases is a different way of defining rare diseases from other countries, representing rare diseases in terms of prevalence or number of patients based on socioeconomic levels. The list is based on the priority criteria of relatively high incidence, high disease burden, and high treatability and includes 121 diseases. The government has also established the Expert Committee of Diagnosis, promoted the establishment of rare diseases clinical research centers, formulated diagnosis and treatment guidelines of rare diseases, and prioritized speeding up the evaluation of drugs for rare diseases [10]. With these efforts, great improvements have been witnessed. For example, the evaluation process of rare disease drugs has been reduced within 3 months. According to incomplete statistics, 95 rare disease drugs have been marketed in China by the end of 2020 [11]. The national health insurance covered the medical costs for rare disease treatment. The national health insurance system consists of two types of programmers: basic medical insurance for urban employees (BMIUE) and basic medical insurance for urban and rural residents (BMIURR). In recent years, adjustments to the National Health Insurance Medicine Catalogue have focused on drugs for rare diseases, with more rare disease drugs included in the catalogue. By the end of 2021, 62 rare disease drugs were included in the National Health Insurance Medicine Catalogue, which were divided into two categories and referred to as ‘Class A’ and ‘Class B’. Fifteen drugs were covered by Class A, which were fully included in the reimbursement scope and are reimbursed according to health insurance rate. Forty-seven drugs were covered by Class B, which required 10–30% out-of-pocket payment before insurance reimbursement and were reimbursed according to health insurance rate [12]. Some regions also pay a portion of the cost of rare disease drugs through severe diseases insurance.

Despite the achievements, it remains to be seen whether the needs of rare disease patients are adequately covered and whether the costs of rare disease drugs are affordable. Previous studies show that rare disease treatments require significant expenditure and may force patients to cease treatments [13,14]. One study found that 22 drugs for 14 rare diseases were unaffordable for most residents in China [15]. Other studies found that most rare disease drugs placed a heavy financial burden on patients with rare diseases, and the affordability of treatment was relatively poor [16,17]. In 2019, a comprehensive social survey of patients with rare diseases in China reported that about 36.8% of patients did not receive treatment mainly because of the significant health expenditure [18]. At present, relevant research mainly focuses on a few rare diseases and drugs in China, and there is a lack of research that reflects the latest achievements of China’s rare disease policies. The objective of this study was to evaluate the price and affordability drugs for rare diseases in Shandong province in 2021, using an adaptation of WHO/HAI methodology [19].

## 2. Materials and Methods

### 2.1. Study Setting

A cross-sectional survey was conducted using standard WHO/HAI methodology in Shandong Province. Shandong is a coastal province in northern China, with a population of 100 million and the third-highest GDP among 31 provinces and regions in Mainland China in 2021 [20]. Shandong Province is one of the first provinces in China to focus on the health care security of rare disease drugs for rare diseases. It had an early start to attaching great importance to the security system for rare disease drugs. In 2012, a city in Shandong province established a special drug list through a severe disease aid system, including recombinant human interferon for injection (β-1b) and Bosentan tablets [21]. In 2014, Shandong province provided basic living expenses for children of low-income families with rare diseases requiring long-term treatment [22]. Health insurance included more than a dozen rare diseases, including hemophilia and pulmonary arterial hypertension in 2015. Drugs used to treat phenylketonuria, Gaucher disease, Pompe disease, and Fabry disease were included in the health insurance drug catalog for severe diseases in recent years [23,24]. As a province with a developed economy, large population, and an early start in rare disease security, Shandong can be a typical case of the affordability of rare disease drugs in China.

### 2.2. Selection of Drugs

First, we collected all the drugs recommended in the Rare Diseases Diagnosis and Treatment Guidelines (2019 Edition) [25], from which the drugs that were marketed in China with rare disease indications were selected. We then selected from them the drugs that were used in secondary and tertiary public hospitals in Shandong province. A list of 50 drugs involving 22 rare diseases was included in this survey.

### 2.3. Data Collection

The survey was supported by the Health Commission of Shandong province. The price data were obtained from the key monitoring secondary and tertiary public hospitals. As rare disease drugs are mainly available in secondary and tertiary hospitals of China [17], the survey data could represent the rare disease drug use in Shandong. The survey period for this study was from January to March 2021. The drug use records (including drug name, dosage forms, unit price, and production enterprises) were collected from the key monitoring secondary and tertiary public hospitals in Shandong Province. Originator Brands, generic drugs, and biosimilars data were obtained for selected rare disease drugs. In addition, the study used the lowest specification as the standard for the drugs under the same generic name, translated the price, and took the median unit price of originator brands (OBs) and lowest price generics and biosimilars (LPGs) as analysis objects.

### 2.4. Data Analysis

#### 2.4.1. Price

Following the WHO/HAI methodology, medicine prices were compared with international reference prices (IRPs) to obtain a median price ratio (MPR). MPR is an important indicator to evaluate the price level of medicines in the survey area, which is an expression of how much greater or less the local medicine price is compared to the IRP [19]. The MPR is calculated as follows:

$$Median\;Price\;Ratio\;\left(MPR\right)=\frac{median\;local\;unit\;price}{international\;reference\;unit\;price}$$

Generally, an MPR of one or less indicates an efficient public sector procurement system [19].

The International Medical Products Price Guide from Management Sciences for Health (MSH) is recommended as the most commonly used reference price by the WHO/HAI methodology. Furthermore, this methodology stated, “Should you choose to use an alternative set of reference prices, you might consider using the New Zealand Pharmaceutical Management Agency (PHARMAC) prices or the Australian Pharmaceutical Benefits Scheme (PBS) prices.” [19]. Based on the above, we used two sets of reference prices as IRPs. One set was MSH, including prices for 11 rare disease drugs surveyed. The other set of reference prices was obtained from PBS, including 34 drugs surveyed. Since our prices came from the hospitals and were not reimbursement prices, we adopted the “dispensed price for maximum quantity” (DPMQ) of PBS as reference prices [26,27]. The MSH reference prices were published in 2015, and the DPMQ were collected in 2022. The prices of rare disease drugs surveyed in 2021 were adjusted for inflation/deflation based on the corresponding year’s consumer price index (CPI) [28] and converted into dollars by purchasing power parity (PPP) over the same period [29]. Only the MPRs of the 11 drugs with MSH reference prices and the 34 drugs with PBS reference prices were discussed in this study.

#### 2.4.2. Affordability

Affordability is assessed as the number of days’ wages needed by the lowest-paid un-skilled government worker (LPGW) to purchase a treatment course or a monthly treatment in case of chronic conditions where therapy continues indefinitely. For a lack of official data on the daily wages of LPGWs in China, we used urban and rural residents’ daily per capita disposable income as a substitute [15,30]. All 22 rare diseases in our survey require long-term or even lifelong medications and the drugs surveyed are maintenance drugs. Based on the Rare Diseases Diagnosis and Treatment Guidelines (2019 Edition) [25], the official drug instructions, literature review [15,31], and expert opinions, affordability is then expressed as the number of days of daily per capita disposable income to purchase a monthly (30 days) treatment of each rare disease drug. If the cost of a monthly treatment of drugs do not exceed the daily per capita disposable income, it is considered affordable. The calculation formula was as follows:

$$Affordability=\frac{Cost\;of\;one-month’\;s\;supply\;of\;orphan\;drugs\;for\;rare\;diseases}{Daily\;per\;capita\;disposable\;income}$$

The daily dose information for rare disease drugs was based on recommendations from the Rare Diseases Diagnosis and Treatment Guidelines (2019 Edition) [25], Defined Daily Dose (DDD) [32], and the official drug instructions. For some rare diseases for which the drug dose is based on the patient’s weight or body surface area, the assumed values were 70 kg for an adult weight and 1.7 m^2^ for a body surface area [33]. As the dose of some rare disease drug treatments may vary depending on the severity of diseases and the stage of treatment, we used the doses of moderate severity and maintenance regimens.

Since the income of urban residents exceeded that of rural residents, per capita disposable income data for both residents were collected separately. In 2021, the daily per capita disposable income of urban residents in Shandong Province was CNY 128.95 (USD 30.85), and the daily per capita disposable income of rural residents was CNY 56.97 (USD 13.63).

We calculated the affordability of out-of-pocket costs for rare disease drugs without health insurance and with health insurance. Since we used the per capita disposable income of urban residents and rural resident instead of the daily wages of LPGWs, only BMIURR was considered (the reimbursement ratio of BMIUE is higher than that of BMIURR, so the drugs are more affordable for urban employees). The design and benefit packages of the BMIURR in each city of Shandong possibly differ, and there is disparity between medical cost and security level of basic health insurance for different rare diseases [34]. We adopted the average reimbursement ratio of hospital expenses as a substitute solution to estimate the actual affordability level of rare disease drugs in Shandong. The reimbursement ratio of BMIURR in Shandong Province in 2020 was used for the analysis. In 2020, the average reimbursement ratio of hospitalization expenses of BMIURR reached 63.2% [35], and this ratio was used for drugs covered by Class A health insurance. For drugs covered by Class B health insurance, we set 30% patient out-of-pocket payment [36] and then calculated drug costs at a 63.2% reimbursement ratio. For drugs included in the scope of severe disease insurance in Shandong, the reimbursement ratio for Saproterin was 60% after an out-of-pocket payment of CNY 20,000 (USD 4784.69). Insurance reimburses up to CNY 900,000 (USD 215,311) for Imiglucerase, Agalsidase beta, and Alglucosidase alfa in a year [24]. We used out-of-pocket spending after health plan reimbursement for patients with rare diseases to calculate the affordability.

Statistical analysis was carried out using SPSSPRO (Version 1.0.11, Suzhou Zhongyan Network Technology Co., Ltd., Suzhou, China). Rare disease drug characteristics were summarized using descriptive statistics. Owing to the non-normal distribution of drug prices and costs of one month’s treatment, non-parametric analysis was used for the statistics. The statistical significance level was set to *p* < 0.05.

## 3. Results

### 3.1. Basic Information of the Surveyed Rare Disease Drugs

This study included 50 drugs involving 22 rare diseases based on China’s First List of Rare Diseases. Of the 50 rare disease drugs, 21 were marketed as originator brands, 16 were marketed as LPGs, and 13 drugs both have originator brands and generic (or biosimilar) versions, totaling 34 OBs and 29 LPGs. Of the 50 rare disease drugs, 19 had indications for rare diseases only and 31 had indications for multiple diseases. Forty surveyed drugs were covered by national health insurance. Nine drugs were covered by Class A health insurance, and thirty-one drugs were covered by Class B health insurance (tranexamic acid injections were covered by Class A while tranexamic acid tablets were covered by Class B). Among the drugs included in national health insurance, 13 drugs had indications only for rare diseases, and the other 27 drugs have multiple disease indications. Of the remaining 10 drugs excluded from the National Health Insurance Medicine Catalogue, Saproterin, Imiglucerase, Agalsidase beta, and Alglucosidase alfa were guaranteed by severe disease insurance in Shandong Province. All of these drugs were originator drugs and were only indicated for rare diseases.

### 3.2. Price

#### 3.2.1. Basic Information on Drug Prices

**Figure 1:**
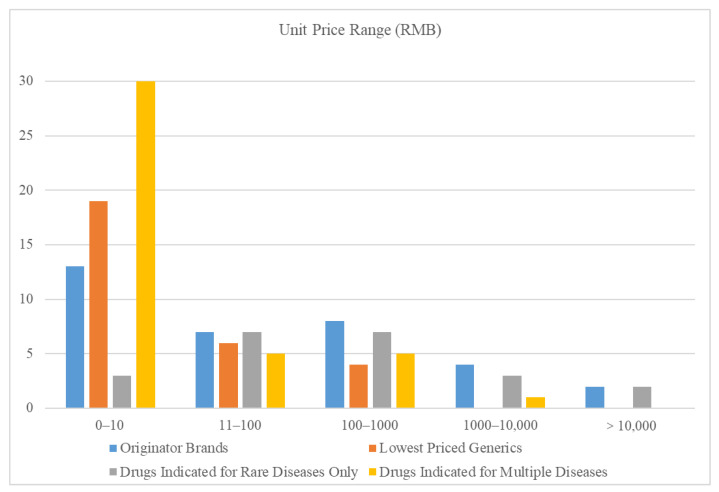
Price distribution of rare disease drugs.

The unit price of 50 rare disease drugs ranged from CNY 0.12 (USD 0.03) to CNY 552,261 (USD 132,119.86) (median CNY 13.69/USD 3.28) (see Table 1). Six rare disease drugs were priced at CNY 1000 or more: Imiglucerase (CNY 20,081.22/USD 4789.05), Nusinersen (CNY 552,261/USD 132,119.86), Emicizumab (CNY 8133.52/USD 1945.75), Agalsidase beta (CNY 5874.05/USD 1405.28), Alglucosidase alfa (CNY 5271.63/USD 1261.16), and Evolocumab (CNY 1299.53/USD 310.89). The unit price of 34 OBs ranged from CNY 1.66 (USD 0.40) to CNY 552,261 (USD 132,119.86) (median CNY 57.1/USD 13.66). The unit price of 29 LPGs ranged from CNY 0.10 (USD 0.02) to CNY 887.50 (USD 212.32) (median CNY 5.25/USD 1.26). The unit price of drugs with indications for rare diseases only ranged from CNY 0.92 (USD 0.22) to CNY 552,261 (USD 132,119.86) (median CNY 132.61/USD 31.72). The unit price of drugs indicated for multiple diseases ranged from CNY 0.10 (USD 0.02) to CNY 1299.53 (USD 310.89) (median CNY 5.25/USD 1.26). The distribution of price is shown in Figure 1. It can be seen that most LPGs and multi-indication drugs were priced between CNY 0 and 10. Drugs with a price higher than CNY 1000 were all OBs and drugs indicated for rare diseases only, and only one multi-indication drug was in this range.

#### 3.2.2. Median Price Ratio (MPR)

Table 1 summarizes the MPRs of drugs with IPRs. Eleven surveyed drugs had MSH reference prices. One generic drug had indication for rare disease only. The MPRs ranged from 0.45 to 32.79 (median 1.33). The prices of 11 drugs were slightly higher than the MSH reference price. The median MPR of 11 LPGs was 0.98, and for two OBs, its value was 20.69. Pyridostigmine was a generic drug with indication for rare disease only, and its MPR was 0.79. The median MPR of the other multi-indication drugs was 1.60. Thirty-four surveyed drugs had PBS reference prices. The MPRs ranged from 0.08 to 7.01 (median 1.97), indicating that the prices of rare disease drugs surveyed were generally higher than the DPMQ reference price. The median MPR of 19 LPGs was 0.80, and for 26 OBs, its value was 2.27. The median MPR of 11 drugs indicated for rare diseases only was 0.88, and for 23 drugs indicated for multiple diseases, its value was 2.10.

### 3.3. Affordability

#### 3.3.1. Affordability of Out-of-Pocket Cost without Health Insurance

Table 2 details the cost of one month’s treatment and the affordability of 50 rare disease drugs surveyed in 2021. The cost of 50 drugs ranged from 0.05 (Atorvastatin, Amantadine) to 4671.94 (Imiglucerase) days of daily per capita disposable income of urban residents, and 0.11 (Atorvastatin, Amantadine) to 10,574.65 (Imiglucerase) days of daily per capita disposable income of rural residents. The median affordability of rare disease drugs was 6.89 for urban residents and 15.60 for rural residents. There were 36 (72.00%) and 44 (88.00%) rare disease drugs difficult to afford for urban and rural residents, respectively, showing that the rare disease drugs were generally unaffordable. Among those unaffordable drugs, Imiglucerase for GD was the highest, the cost of which was CNY 602,436.6 (USD 114,123.6), equivalent to 4671.94 days of daily per capita disposable income of urban residents and 10,574.65 days of daily per capita disposable income of rural residents.

Only three OBs—Levodopa and Benserazide Hydrochloride, Carbidopa and Levodopa, and Selegiline for EOPD and YOPD—were affordable by urban residents, and no OBs was affordable by rural residents. The cost of 34 OBs ranged from 0.70 (Carbidopa and Levodopa) to 4671.94 (Imiglucerase) days of daily per capita disposable income of urban residents and 1.57 (Carbidopa and Levodopa) to 10,574.65 (Imiglucerase) days of daily per capita disposable income of rural residents. The median affordability of OBs was 25.90 for urban residents and 58.62 for rural residents. The top five unaffordable OBs were Imiglucerase, Agalsidase beta, Alglucosidase alfa, Nusinersen, and Emicizumab, the costs of which were more than 900 days’ income for urban residents and 2000 days’ income for rural residents.

Twelve and six LPGs were affordable for urban and rural residents, respectively. The cost of 29 LPGs ranged from 0.05 (Atorvastatin, Amantadine) to 154.17 (Recombinant human coagulation factor VIII) days of daily per capita disposable income of urban residents and from 0.11 (Atorvastatin, Amantadine) to 348.96 (Recombinant human coagulation factor VIII) days of daily per capita disposable income of rural residents. The median affordability of LPGs was 3.73 for urban residents and 8.44 for rural residents. The top five most unaffordable LPGs were Recombinant human coagulation factor VIII, Human coagulation factor VIII, Pirfenidone, Human Immunoglobulin, and Human prothrombin complex.

The Mann–Whitney U test compared the cost of one month’s treatment for OBs and LPGs. There was a significant statistical difference between the two types (*p* = 0.001). (See Table 3.)

Of the 19 drugs that had indications for rare diseases only, one drug, Pyridostigmine for CMS, was affordable for urban residents, and none were affordable for rural residents. The cost of 19 drugs had indications for rare diseases only ranged from 0.64 (Pyridostigmine) to 4671.94 (Imiglucerase) days of daily per capita disposable income of urban residents and from 1.45 (Pyridostigmine) to 10,574.65 (Imiglucerase) days of daily per capita disposable income of rural residents. The median affordability of drugs indicated for rare diseases only was 70.42 for urban residents and 159.40 for rural residents. Of the 31 drugs for multiple diseases, 13 and 6 are affordable for urban and rural residents, respectively. The cost of 31 drugs indicated for multiple diseases ranged from 0.05 (Atorvastatin, Amantadine) to 88.77 (Recombinant Human Granulocyte Colony-stimulating Factor) days of daily per capita disposable income of urban residents and from 0.11 (Atorvastatin, Amantadine) to 200.92 (Recombinant Human Granulocyte Colony-stimulating Factor) days of daily per capita disposable income of rural residents. The median cost of drugs for commonly encountered diseases was CNY 305.70 (USD 73.13), equivalent to 2.37 days and 5.37 days of daily per capita disposable income of urban and rural residents, respectively. There was a statistically significant increase in the affordability of drugs for the commonly encountered disease compared to the drugs with indications for rare diseases only (*p* = 0.001, Mann–Whitney U test). (See Table 3).

#### 3.3.2. Affordability of Out-of-Pocket Cost with Health Insurance

All generic drugs were covered by national health insurance, except sildenafil. The drugs excluded from the national health insurance were all OBs. The number of affordable drugs increased from 14 to 16 for urban residents and from 6 to 12 for rural residents using the health insurance system, and there were still 34 (68.00%) and 38 (78.00%) drugs beyond affordability for them. The out-of-pocket costs of 50 drugs ranged from 0.02 (Atorvastatin, Amantadine) to 4090.31 (Imiglucerase) days of daily per capita disposable income of urban residents and from 0.04 (Atorvastatin, Amantadine) to 9258.17 (Imiglucerase) days of daily per capita disposable income of rural residents. The median cost was 3.84 days and 8.70 days of daily per capita disposable income of urban and rural residents, respectively.

Urban residents could afford 6 OBs and 14 LPGs, while rural residents could afford 3 OBs and 10 LPGs after reimbursement. The cost of 34 OBs ranged from 0.30 (Carbidopa and Levodopa) to 4090.31 (Imiglucerase) days of daily per capita disposable income of urban residents and 0.67 (Levodopa and Benserazide Hydrochloride) to 9258.17 (Imiglucerase) days of daily per capita disposable income of rural residents. The median affordability of OBs was 14.94 for urban residents and 33.37 for rural residents after reim- bursement. The cost of 29 LPGs ranged from 0.02 (Atorvastatin, Amantadine) to 85.97 (Recombinant human coagulation factor VIII) days of daily per capita disposable income of urban residents and from 0.05 (Amantadine) to 194.58 (Recombinant human coagulation factor VIII) days of daily per capita disposable income of rural residents. The median af- fordability of LPGs was 2.08 for urban residents and 4.70 for rural residents after reim-bursement. There was a significant statistical difference between the cost of one month’s treatment for OBs and LPGs after reimbursement (*p* = 0.001, Mann–Whitney U test). (See Table 3).

Pyridostigmine indicated for rare disease only was affordable for urban residents and rural residents after reimbursement. The cost of 19 drugs indicated for rare disease only ranged from 0.24 (Pyridostigmine) to 4090.31 (Imiglucerase) days of daily per capita disposable income of urban residents and from 0.53 (Pyridostigmine) to 9258.17 (Imiglucerase) days of daily per capita disposable income of rural residents. The median affordability of drugs indicated for rare diseases only was 33.49 for urban residents and 75.81 for rural residents. Of the 31 drugs for multiple diseases, 15 and 11 were affordable for urban and rural residents after reimbursement. The cost of 31 drugs indicated for multiple diseases ranged from 0.02 (Amantadine) to 55.53 (Highly purified menotrophin) days of daily per capita disposable income of urban residents and from 0.05 (Amantadine) to 125.70 (Highly purified menotrophin) days of daily per capita disposable income of rural residents. The median affordability of drugs indicated for multiple diseases was 1.27 for urban residents and 2.87 for rural residents. There was a significant statistical difference between the costs of one month’s treatment for these two groups of drugs after reimbursement (*p* < 0.001, Mann–Whitney U test). (See Table 3.)

## 4. Discussion

This study conducted a cross-sectional survey of data from secondary and tertiary public hospitals in Shandong province to estimate the price and affordability of rare disease drugs using an adaptation of WHO/HAI methodology. The prices of some drugs were higher than MSH prices, even higher than prices of developed country, meaning the medical costs are high and the affordability was low for patients with rare diseases. Similar trends were observed in previous findings [15,16,17].

Chinese patients have to pay higher costs for the same rare disease drugs, and pharmaceutical spending is a relatively large proportion of per capita income. Actions have been taken to lower the price of rare disease drugs in China, such as reducing the value-added tax to 3% for rare disease drugs, establishing a price negotiation mechanism, and centralized purchasing [37]. However, these measures cover a limited number of rare disease drugs.

It is necessary to develop pricing mechanisms for rare disease drugs, especially for the relatively high-priced originator drugs. The government can negotiate differential prices with manufacturers of rare disease drugs by including drugs in the coverage of national or provincial government-sponsored insurance or by buying drugs in bulk to lower prices. There is a need to establish a unique evaluation process of pricing and reimbursement for rare disease drugs using new assessment techniques. Previous studies have shown that using health technology assessment (HTA) approaches typically results in rare disease drugs not being cost-effective [38,39,40]. The multi-criteria decision analysis (MCDA) framework as a tool for assessing the value of rare disease drugs has improved the technical and ethical quality of decisions regarding prioritization, coverage, and reimbursement of rare diseases [41,42,43].

Setting rare disease drug prices using external reference pricing (ERP) will be convincing evidence when purchasing departments negotiate with the manufacturer [44]. The scientific selection of a basket of reference countries is necessary. Similarities with the economics, purchasing power, population, age distribution, and incidence of rare diseases are the main factors to be considered in selecting these reference countries. There are no countries with high similarity to China that can be directly selected as a reference country. Countries with similar levels of economic development, such as India, Brazil, and South Africa, can be used as external reference pricing countries. It is also necessary to consider some developed countries with more stringent price regulations, faster launch of new drugs, and stable drug prices, such as the UK, Australia, and Japan. In the actual pricing, different weights can be used to reflect the different levels of economic development [45].

Compared with generic drugs and biosimilars, originator drugs were less affordable. High-quality generic drugs or biosimilars can replace originator drugs, and increased competition from manufacturers may also decrease the prices of rare disease drugs [46,47]. The government should improve policies to support generics and biosimilars such as encouraging R&D of these types of drugs, facilitating generic drug consistency evaluation, and giving priority to review and approval to accelerate market access.

Drugs that had indications for rare diseases only were less affordable than those for multiple diseases. Of the drugs surveyed, the median unit price for drugs with rare dis-ease indications was much higher than that of drugs with multiple indications. The possible explanation for this is that the price of rare disease drug is related to its prevalence [48]. Although 11 drugs indicated for rare disease only were available at price levels lower than PBS price, they were still unaffordable for Chinese patients. Even after health insurance reimbursements, almost all the drugs with indication for rare disease only were still unaffordable for urban and rural resident. In China, the health insurance system prefers to include more drugs indicated for multiple diseases because such drugs can be used to treat various diseases and more patients, including rare diseases. Repurposed or repositioned drugs have been used to treat rare diseases and to significantly improve the affordability of rare disease drugs [49,50]. Some drugs with multiple indications can treat rare diseases, although off-label. Repurposing these drugs is a faster and cheaper way to develop rare disease drugs.

The results of this investigation showed that the healthcare security system had positive effects but was insufficient to alter the affordability of rare disease drugs. Future efforts should improve the health security system for patients with rare diseases. More patients with rare diseases can rely on multiple fundraising sources to afford rare disease drugs, including commercial insurance, medical assistance, and social charity. Rare diseases charity funds have provided several forms of help to domestic rare diseases patients in past years [10]. Commercial health insurance as supplementary to the basic schemes should be better used to improve medication coverage for high-value rare disease drugs. If necessary, the government can take measures such as legislation prohibiting commercial insurers from including rare diseases in exclusion clauses [51].

The income gap was large between urban and rural residents. To improve the affordability of rare disease drugs, the redistribution of income to rural low-income groups in rural areas through a healthcare security system is necessary. Such policies can include reducing or waiving individual financing, increasing medical assistance to the low-income group, expanding the scope of assistance, and providing bottom-up protection for rural patients with rare diseases.

The present study has several limitations. First, our data may not fully capture all rare disease drug use in China because the selection of drugs and diseases was according to the China’s First National Rare Disease List and the survey was mainly based Shandong province. Second, the MSH 2015 price and DPMQ of PBS in Australia were selected as the IRPs, and we only discussed the MPRs of some surveyed rare disease drugs. The MPR results with Australian PBS prices as reference may affect the accuracy of price estimates. Moreover, for some drugs, hospital prices may be lower than community pharmacy in Australia. Third, to facilitate the assessment of affordability, all rare disease drugs were calculated as the cost of one month of treatment, which may cause bias in affordability. Last, the study focuses on the affordability of drugs, but the treatment costs of patients with rare diseases include transportation costs and nutrition costs incurred during medical visits or treatment. The actual economic burden of patients with a rare disease can be even heavier. Despite these limitations, our data suggest that the affordability of rare disease drugs in Shandong province is limited.

## 5. Conclusions

The results showed that the prices of some rare disease drugs were higher compared with international reference prices, and most drugs remain unaffordable for patients with rare diseases in Shandong, China. This highlights the need for a range of government policies to ensure that rare diseases are affordable. It is recommended that government reduce the prices of rare disease drugs by developing the pricing mechanisms, negotiating with manufacturers, encouraging the development of generics and biosimilars, and promoting the repurposing of drugs. The healthcare security system of rare diseases needs further improvement through using multiple sources of fund-raising and strengthening assistance for rural patients.

## Figures and Tables

**Table 1:** General information and MPR on 50 rare disease drugs surveyed.

Condition	Medicine Name	Type ^a^	Indications for Rare Diseases Only ^b^	Dosage Form	Minimum Strength	Median Unit Price (CNY)	MPR (MSH) ^c^	MPR (PBS) ^c^
Amyotrophic Lateral Sclerosis	Edaravone	LPGs	N	injection	5 mL:10 mg	8.44	NA	NA
	Riluzole	OBs	Y	tablets	50 mg	68.46	NA	7.01
		LPGs	Y	tablets	50 mg	25.00	NA	2.56
Primary Carnitine Deficiency	Levocarnitine	LPGs	Y	oral solution	10 mL:1 g	8.01	NA	NA
Adrenal Hypoplasia Congenita	Hydrocortisone	LPGs	N	tablets	20 mg	0.97	3.48	0.68
Congenital Myasthenic Syndrome	Pyridostigmine	LPGs	Y	tablets	60 mg	0.92	0.79	0.88
Fabry disease	Agalsidase beta	OBs	Y	injection	5 mg	5874.05	NA	NA
Gaucher Disease	Imiglucerase	OBs	Y	injection	400 IU	20,081.22	NA	NA
Glycogen Storage DiseaseⅠ, Glycogen Storage DiseaseⅡ	Alglucosidase alfa	OBs	Y	injection	50 mg	5271.63	NA	NA
Hemophilia	Recombinant human coagulation factor Ⅷ	OBs	Y	injection	250 IU	999.65	NA	NA
		LPGs	Y	injection	250 IU	887.50	NA	NA
	Human coagulation factor Ⅷ	LPGs	Y	injection	200 IU	378.65	NA	NA
	Human prothrombin complex	LPGs	Y	injection	200 IU	177.79	NA	NA
	Emicizumab	OBs	Y	injection	30 mg	8133.25	NA	NA
Hepatolenticular degeneration	Penicillamine	LPGs	N	tablets	0.125 g	0.79	0.68	0.48
Hereditary Angioedema	Danazol	LPGs	N	capsules	0.1 g	1.02	0.61	NA
	Tranexamic acid	OBs	N	tablets	0.5 g	2.02	NA	2.04
		LPGs	N	injection	0.25 g	5.25	0.45	NA
Homozygous Familial Hypercholesterolemia	Ezetimibe	OBs	N	tablets	10 mg	6.89	NA	3.75
		LPGs	N	tablets	10 mg	6.74	NA	3.67
	Evolocumab	OBs	N	injection	1 mL:0.14 g	1299.53	NA	2.10
	Rosuvastatin	OBs	N	tablets	10 mg	5.55	NA	4.04
		LPGs	N	tablets	5 mg	0.10	NA	0.08
	Atorvastatin	OBs	N	tablets	10 mg	3.33	32.79	2.73
		LPGs	N	tablets	10 mg	0.10	0.98	0.08
Hyperphenylalaninemia	Sapropterin hydrochloride	OBs	Y	tablets	0.1 g	8.33	NA	0.11
Idiopathic Hypogonadotropic Hypogonadism	Highly purified meMtrophin	OBs	N	injection	75 IU	238.70	NA	NA
	MeMtrophin	LPGs	N	injection	75 IU	19.08	NA	NA
	Recombinant human choriogonadotropin	OBs	N	injection	250 μg: 0.5 mL	192.97	NA	1.25
	Chorionic gonadotrophin	LPGs	N	injection	1000 IU	6.43	0.61	0.88
Idiopathic Pulmonary Arterial Hypertension	Sildenafil	OBs	N	tablets	0.1 g	81.93	NA	3.89
		LPGs	N	tablets	25 mg	1.58	NA	0.10
	Ambrisentan	OBs	Y	tablets	5 mg	61.20	NA	0.37
		LPGs	Y	tablets	5 mg	22.34	NA	0.13
	Bosentan	OBs	Y	tablets	0.125 g	53.00	NA	2.66
	Selexipag	OBs	Y	tablets	0.2 mg	47.37	NA	0.31
	Macitentan	OBs	Y	tablets	10 mg	138.01	NA	0.57
Idiopathic Pulmonary Fibrosis	Nintedanib	OBs	Y	capsules	0.15 g	127.20	NA	0.86
	Pirfenidone	LPGs	Y	capsules	0.1 g	13.69	NA	1.22
Multiple Sclerosis	Baclofen	OBs	N	tablets	10 mg	2.53	8.59	4.37
		LPGs	N	tablets	10 mg	1.26	4.28	2.18
	TerifluMmide	OBs	Y	tablets	14 mg	283.15	NA	6.07
Young-Onset Parkinson’s Disease, Early Onset Parkinson’s Disease	Piribedil	OBs	N	tablets	50 mg	2.44	NA	NA
	Levodopa and Benserazide Hydrochloride	OBs	N	tablets	0.25 g	1.93	NA	1.68
	Carbidopa and Levodopa	OBs	N	tablets	0.25 g	1.66	NA	0.93
	Compound Carbidopa	LPGs	N	tablets	0.275 g	2.47	2.53	2.50
	Entacapone, Levodopa and Carbidopa	OBs	N	tablets	0.325 g	7.67	NA	2.27
	Rasagiline	OBs	N	tablets	1 mg	40.00	NA	5.28
	Selegiline	OBs	N	tablets	5 mg	3.21	NA	2.26
		LPGs	N	tablets	5 mg	2.89	NA	2.04
	Pramipexole	OBs	N	tablets	0.25 mg	5.45	NA	6.93
		LPGs	N	tablets	0.25 mg	2.63	NA	3.35
	Ropinirole	LPGs	N	tablets	0.5 mg	8.17	NA	NA
	Trihexyphenidyl	LPGs	N	tablets	2 mg	0.18	1.88	0.73
	Amantadine	LPGs	N	tablets	0.1 g	0.12	NA	0.12
	Rotigotine	OBs	N	patches	9 mg: 20 cm^2^	38.16	NA	3.14
Primary Combined ImmuMdeficiency	Human ImmuMglobulin (pH4)	LPGs	N	injection	1.25 g	273.63	1.33	NA
Progressive Familial Intrahepatic Cholestasis	Ursodeoxycholic acid	OBs	N	capsules	0.25 g	8.41	NA	2.83
		LPGs	N	tablets	50 mg	1.76	NA	0.23
Severe Congenital Neutropenia	Recombinant Human Granulocyte Colony–stimulating Factor	OBs	N	injection	0.1 mg	272.53	NA	NA
		LPGs	N	injection	75 µg	31.74	NA	NA
Spinal Muscular Atrophy	Nusinersen	OBs	Y	injection	5 mL:12 mg	552,261.00	NA	1.91
Tuberous Sclerosis Complex	Everolimus	OBs	N	tablets	5 mg	130.01	NA	1.65

^a^ OBs = Originator Brands; LPGs (Lowest Priced Generics); ^b^ Y = Yes N = No; ^c^ NA = Not Available.

**Table 2:** Affordability of 50 rare disease drugs surveyed.

Medicine Name	Type ^a^	Indications for Rare Diseases Only ^b^	Total Usage per Month	Health Insurance Catalogue	Days of Daily per Capita Disposable Income							
					OOP without Health Insurance				OOP with Health Insurance			
					Urban Residents	Afforda-bility ^c^	Rural Residents	Afforda-bility ^c^	Urban Residents	Afforda-bility ^c^	Rural Residents	Afforda-bility ^c^
Edaravone	LPGs	N	60 mg × 14	Class B	5.50	N	12.44	N	3.07	N	6.94	N
Riluzole	OBs	Y	0.1 g × 30	Class B	31.85	N	72.10	N	17.76	N	40.20	N
	LPGs	Y	0.1 g × 30	Class B	11.63	N	26.33	N	6.49	N	14.68	N
Levocarnitine	LPGs	Y	2 g × 30	Class B	3.73	N	8.44	N	2.08	N	4.70	N
Hydrocortisone	LPGs	N	30 mg × 30	Class A	0.34	Y	0.77	Y	0.12	Y	0.28	Y
Pyridostigmine	LPGs	Y	0.18 g × 30	Class A	0.64	Y	1.45	N	0.24	Y	0.53	Y
Agalsidase beta ^c^	OBs	Y	5 mg × 30	No	1366.61	N	3093.24	N	784.98	N	1776.76	N
Imiglucerase ^c^	OBs	Y	300 IU × 30	No	4671.94	N	10,574.65	N	4090.31	N	9258.17	N
Alglucosidase alfa ^c^	OBs	Y	0.1 g × 30	No	1226.46	N	2776.01	N	644.83	N	1459.52	N
Recombinant human coagulation factor Ⅷ	OBs	Y	700 IU × 8	Class B	173.65	N	393.05	N	96.83	N	219.17	N
	LPGs	Y	700 IU × 8	Class B	154.17	N	348.96	N	85.97	N	194.58	N
Human coagulation factor Ⅷ	LPGs	Y	700 IU × 8	Class A	82.22	N	186.10	N	30.26	N	68.49	N
Human prothrombin complex	LPGs	Y	700 IU×8	Class B	38.61	N	87.38	N	21.53	N	48.72	N
Emicizumab	OBs	Y	15 mg × 30	No	946.11	N	2141.46	N	946.11	N	2141.46	N
Penicillamine	LPGs	N	0.5 g × 30	Class A	0.74	Y	1.66	N	0.27	Y	0.61	Y
Danazol	LPGs	N	0.2 g × 30	Class B	0.47	Y	1.07	N	0.26	Y	0.60	Y
Tranexamic acid	OBs	N	2 g × 30	Class B	1.88	N	4.25	N	1.05	N	2.37	N
	LPGs	N	2 g × 30	Class A	9.77	N	22.12	N	3.60	N	8.14	N
Ezetimibe	OBs	N	10 mg × 30	Class B	1.60	N	3.63	N	0.89	Y	2.02	N
	LPGs	N	10 mg × 30	Class B	1.57	N	3.55	N	0.87	Y	1.98	N
Evolocumab	OBs	N	420 mg × 1	Class B	30.23	N	68.43	N	16.86	N	38.15	N
Rosuvastatin	OBs	N	10 mg × 30	Class B	1.29	N	2.92	N	0.72	Y	1.63	N
	LPGs	N	10 mg × 30	Class B	0.05	Y	0.11	Y	0.03	Y	0.06	Y
Atorvastatin	OBs	N	20 mg × 30	Class B	1.55	N	3.51	N	0.86	Y	1.96	N
	LPGs	N	20 mg × 30	Class B	0.05	Y	0.11	Y	0.03	Y	0.06	Y
Sapropterin hydrochloride ^c^	OBs	Y	1400 mg × 30	No	27.13	N	61.41	N	18.61	N	42.12	N
Highly purified menotrophin	OBs	N	75 IU × 30	No	55.53	N	125.70	N	55.53	N	125.70	N
Menotrophin	LPGs	N	75 IU × 30	Class B	4.44	N	10.05	N	2.48	N	5.60	N
Recombinant human choriogonadotropin alfa solution	OBs	N	250 IU × 30	No	44.89	N	101.62	N	44.89	N	101.62	N
Chorionic gonadotrophin	LPGs	N	250 IU × 30	Class A	0.37	Y	0.85	Y	0.14	Y	0.31	Y
Sildenafil	OBs	N	60 mg × 30	No	11.44	N	25.89	N	11.44	N	25.89	N
	LPGs	N	60 mg × 30	No	0.88	Y	2.00	N	0.88	Y	2.00	N
Ambrisentan	OBs	Y	7.5 mg × 30	Class B	21.36	N	48.34	N	11.91	N	26.96	N
	LPGs	Y	7.5 mg × 30	Class B	7.80	N	17.65	N	4.35	N	9.84	N
Bosentan	OBs	Y	0.25 g × 30	Class B	24.66	N	55.82	N	13.75	N	31.12	N
Selexipag	OBs	Y	1.8 mg × 30	Class B	99.19	N	224.50	N	55.31	N	125.18	N
Macitentan	OBs	Y	10 mg × 30	Class B	32.11	N	72.68	N	17.90	N	40.52	N
Nintedanib	OBs	Y	0.38 g × 30	Class B	74.97	N	169.69	N	41.80	N	94.62	N
Pirfenidone	LPGs	Y	2.4 g × 30	Class B	76.44	N	173.02	N	42.62	N	96.47	N
Baclofen	OBs	N	50 mg × 30	Class B	2.94	N	6.66	N	1.64	N	3.71	N
	LPGs	N	50 mg × 30	Class B	1.47	N	3.32	N	0.82	Y	1.85	N
Teriflunomide	OBs	Y	14 mg × 30	Class B	65.88	N	149.11	N	36.73	N	83.14	N
Piribedil	OBs	N	0.2 g × 30	Class B	2.27	N	5.14	N	1.27	N	2.87	N
Levodopa and Benserazide Hydrochloride	OBs	N	0.45 g × 30	Class A	0.81	Y	1.83	N	0.30	Y	0.67	Y
Carbidopa and Levodopa	OBs	N	0.45 g × 30	Class B	0.70	Y	1.57	N	0.39	Y	0.88	Y
Compound Carbidopa	LPGs	N	0.45 g × 30	Class B	0.94	Y	2.13	N	0.52	Y	1.19	N
Entacapone, Levodopa and Carbidopa	OBs	N	0.45 g × 30	Class B	2.47	N	5.59	N	1.38	N	3.12	N
Rasagiline	OBs	N	1 mg × 30	Class B	9.31	N	21.06	N	5.19	N	11.75	N
Selegiline	OBs	N	5 mg × 30	Class B	0.75	Y	1.69	N	0.42	Y	0.94	Y
	LPGs	N	5 mg × 30	Class B	0.67	Y	1.52	N	0.37	Y	0.85	Y
Pramipexole	OBs	N	2.5 mg × 30	Class B	12.68	N	28.70	N	7.07	N	16.00	N
	LPGs	N	2.5 mg × 30	Class B	6.12	N	13.85	N	3.41	N	7.72	N
Ropinirole	LPGs	N	6 mg × 30	Class B	22.81	N	51.63	N	12.72	N	28.79	N
Trihexyphenidyl	LPGs	N	10 mg × 30	Class A	0.21	Y	0.47	Y	0.08	Y	0.17	Y
Amantadine	LPGs	N	0.2 g × 30	Class A	0.06	Y	0.13	Y	0.02	Y	0.05	Y
Rotigotine	OBs	N	6 mg × 30	No	5.92	N	13.40	N	5.92	N	13.40	N
Human Immunoglobulin (pH4)	LPGs	N	31.5 g × 1	Class B	53.47	N	121.04	N	29.82	N	67.49	N
Ursodeoxycholic acid	OBs	N	0.75 g × 30	Class A	5.87	N	13.29	N	2.16	N	4.89	N
	LPGs	N	0.75 g × 30	Class A	6.14	N	13.90	N	2.26	N	5.12	N
Recombinant Human Granulocyte Colony–stimulating Factor	OBs	N	0.14 mg × 30	Class B	88.77	N	200.92	N	49.50	N	112.03	N
	LPGs	N	70 µg × 30	Class B	6.89	N	15.60	N	3.84	N	8.70	N
Nusinersen	OBs	Y	0.1 mg × 30	No	1070.71	N	2423.48	N	1070.71	N	2423.48	N
Everolimus	OBs	N	10 mg × 30	Class B	60.49	N	136.93	N	33.73	N	76.35	N

^a^ OBs = Originator Brands; LPGs (Lowest Priced Generics) ^b^ and ^c^ Y = Yes N = No.

**Table 3:** Median cost of a monthly treatment of rare disease drugs (CNY).

Categories	OOP ^a^ without Insurance	*p* ^b^	OOP with Insurance	*p* ^b^
OBs vs. LPGs				
OBs	3339.30	0.001	1901.18	<0.001
LPGs	480.00		267.98	
Indications for rare diseases only vs. Indications for multiple diseases				
Indications for rare diseases only	292.80	0.001	163.27	<0.001
Indications for multiple diseases	9080.85		4319.07	

^a^ OOP = Out Of Pocket; ^b^ The *p*-value for the difference between two categories was determined with the Mann–Whitney U-test.

## Data Availability

All data generated or analyzed during this study are included in this article.
